# Regulation of ferroptosis by BAP1

**DOI:** 10.1038/s41418-026-01661-5

**Published:** 2026-01-23

**Authors:** Kalidou Ali Boubacar, Hind Kahalerras, El Bachir Affar

**Affiliations:** 1 Laboratory for Cell Signaling and Cancer, Maisonneuve-Rosemont Hospital Research Center, CIUSSS de l’Est-de-l’Île-de-Montréal, Montréal, QC Canada; 2 Department of Medicine, University of Montréal, Montréal, QC Canada

**Keywords:** Lipidomics, Membrane lipids, Cell biology, Epigenetics

BRCA1-Associated Protein 1 (BAP1) coordinates both apoptotic and ferroptotic cell death fates through multiple mechanisms. In this issue of Cell Death and Differentiation, Fan et al. show that BAP1 upregulates ACSL4 (long-chain acyl-CoA synthetase 4), increasing incorporation of polyunsaturated fatty acids into phospholipids and amplifying lipid peroxidation, which culminates in ferroptosis. These findings situate ACSL4 as a key effector within the BAP1 signaling network and highlight how a chromatin-linked deubiquitinase can pivotally rewire lipid metabolism to promote ferroptosis [1].

BAP1 is a major tumor suppressor frequently mutated in various cancers [2, 3]. The relevance of BAP1 to cancer pathogenesis is also reflected by the fact that this gene is probably the most frequently mutated gene-encoding deubiquitinase in human malignancies [3]. However, the precise mechanisms through which BAP1 contributes to tumor suppression remain incompletely understood. BAP1 orchestrates chromatin-associated processes by removing monoubiquitination from lysine 119 of histone H2A (H2AK119ub), a modification involved in transcriptional repression and DNA repair [4–6]. Its enzymatic activity underlies multiple biological functions, including, cell proliferation, metabolism, differentiation, embryonic development, and apoptosis [3]. The tumor-suppressive function of BAP1 is therefore believed to result from the cooperation of multiple roles ranging from the maintenance of genomic integrity to the regulation of cell proliferation, differentiation and cell death.

**Fig. 1:**
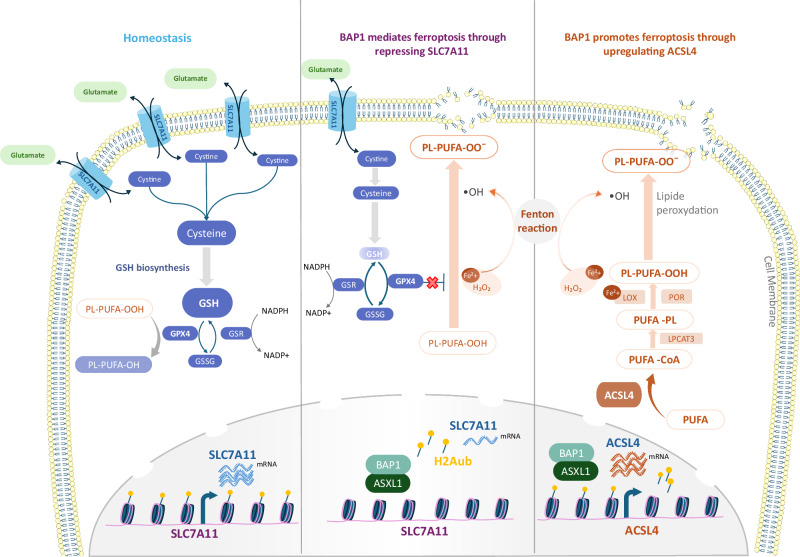
BAP1 promotes ferroptosis through different metabolic pathways. Under homeostatic conditions, cells maintain an effective antioxidant defense system. However, in specific cellular contexts, BAP1 disrupts this balance by repressing SLC7A11 expression through the deubiquitination of H2AK119ub at its promoter, which limits cystine uptake and reduces glutathione (GSH) synthesis. As a consequence, GPX4 activity becomes impaired, weakening the cellular antioxidant defense system. In the presence of polyunsaturated fatty acids (PUFAs), lipid peroxides accumulate within cellular membranes, ultimately triggering membrane rupture and ferroptotic cell death. In addition, BAP1 promotes ferroptosis by upregulating ACSL4 (acyl-CoA synthetase long-chain family member 4). ACSL4 catalyzes the esterification of CoA to specific PUFAs, facilitating their incorporation into membrane phospholipids. These PUFA-containing phospholipids are highly susceptible to peroxidation, thereby increasing cellular sensitivity to ferroptosis. ACSL4 long-chain acyl-coenzyme A (CoA) synthetase 4, ASXL1 Additional Sex combs Like 1, BAP1 BRCA1-Associated Protein 1, GPX4 Glutathione Peroxidase 4, GSR Glutathione Reductase, GSH reduced glutathione, GSSG glutathione disulfide, LOX Lipoxygenase, LPCAT3 Lysophosphatidylcholine Acyltransferase 3, NADP⁺ Nicotinamide Adenine Dinucleotide Phosphate (oxidized form), NADPH Nicotinamide Adenine Dinucleotide Phosphate (reduced form), POR NADPH–cytochrome P450 Oxidoreductase, PL-PUFA-OOH Phospholipid Polyunsaturated Fatty Acid Hydroperoxide, PUFA Polyunsaturated Fatty Acid, PUFA-PL PUFA-containing Phospholipid, SLC7A11 Solute Carrier Family 7 Member 11.

Previous work indicated that BAP1 regulates cell death by ferroptosis, which may account for its tumor-suppressive role [7]. Ferroptosis is a non-apoptotic form of regulated cell death that can be induced by several stimuli including cystine depletion. Cystine serves as a precursor for the synthesis of glutathione (GSH), which is essential for the enzymatic activity of glutathione peroxidase 4 (GPX4), an enzyme responsible for reducing lipid peroxides. Depletion of cystine, particularly through inhibition of the SLC7A11 subunit of the cystine/glutamate antiporter, leads to GPX4 decommissioning and accumulation of lipid peroxides that damage and rupture the plasma membrane, resulting in cell death [8]. This process has been initially termed ferroptosis because the formation of hydroxyl radicals that oxidize lipids is iron-dependent (Fig. 1). The authors have initially presented evidence that BAP1 represses the expression of SLC7A11 by deubiquitinating H2AK119ub at its promoter [7]. This repression leads to intracellular cystine and GSH depletion, resulting in the accumulation of lipid peroxides and subsequent ferroptosis. The authors thus linked BAP1’s tumor-suppressive function, at least in part, to its ability to regulate ferroptosis. Nevertheless, it remained unclear how SLC7A11 is repressed and whether SLC7A11 repression and cystine depletion are the only mechanisms through which BAP1 promotes ferroptosis. Building further on these studies, Fan et al. now show that BAP1 regulates ferroptosis by modulating the expression of the isozyme ACSL4 [1], which catalyzes the conversion of long-chain free fatty acids (PUFA) into acyl-CoA esters, whose peroxidation was previously reported to trigger this form of cell death [9]. The authors first demonstrated that re-expression of BAP1 in BAP1-deficient UMRC6 cells decreases H2AK119ub levels and globally enhances chromatin accessibility at gene bodies and promoters. By integrating multi-omics data from RNA-seq, ATAC-seq, and ChIP-seq analyses, the authors found that BAP1 re-expression modifies the expression of hundreds of genes. Gene ontology analysis revealed that these genes are enriched in pathways related to metabolism, including fatty acid biosynthesis, oxidative stress response, and ferroptosis. These effects were dependent on the catalytic activity of BAP1, as re-expression of the catalytically inactive mutant BAP1^C91A^ did not reproduce these changes.

The authors next investigated whether the observed metabolic alterations induced by BAP1 re-expression could affect ferroptosis. Among the genes identified, *SLC7A11* was already known to modulate ferroptosis [7]. To test whether BAP1-induced ferroptosis depends solely on SLC7A11 regulation, BAP1 was re-expressed in *SLC7A11*-KO UMRC6 cells, where it still promoted ferroptosis, suggesting both SLC7A11-dependent and -independent mechanisms. To identify SLC7A11-independent mechanisms, they focused on ACSL4, which is upregulated upon BAP1 re-expression. Given that ferroptosis results from lipid peroxidation, enzymes such as ACSL4 likely play key roles in this process [8–10]. The authors confirmed that re-expression of BAP1, but not the catalytic dead mutant BAP1^C91A^, reduces H2AK119ub and increases chromatin accessibility at the *ACSL4* promoter and gene body, leading to enhanced ACSL4 mRNA and protein expression without affecting its protein stability. Conversely, BAP1 knockdown reduced *ACSL4* expression and decreased cellular sensitivity to ferroptosis. Moreover, overexpression of ACSL4 in BAP1-knockout 786-O cells restored ferroptotic sensitivity, whereas *ACSL4*-KO cells overexpressing BAP1 were resistant compared to controls. Next, lipidomic analyses revealed that BAP1 increased the levels of several lipid subclasses, including triacylglycerols (TG), phosphatidylcholines (PC), and phosphatidylethanolamines (PE), and this effect was abolished in *ACSL4*-KO cells, indicating that BAP1-mediated lipid metabolism regulation depends on ACSL4. Cancer genomics data analysis further showed a positive correlation between *BAP1* and *ACSL4* expression across multiple cancer types, with both genes being downregulated in tumors compared to normal tissues. Since BAP1 functions as part of the Polycomb Repressive Deubiquitinase (PR-DUB) complex, comprising cofactors such as FOXK1/2, KDM1B, OGT, HCFC1, and ASXL1/2 [11–13], the authors also examined the roles of these proteins in ACSL4 regulation and ferroptosis. Mutations disrupting BAP1 interactions with these partners revealed that only ASXL1/2 significantly influenced ACSL4 upregulation, and cellular sensitivity to ferroptosis. Notably, BAP1 overexpression in ASXL1/2-KO cells failed to induce ACSL4 expression or sensitize cells to ferroptosis.

In conclusion, BAP1 appears to regulate ferroptotic cell death through two mechanisms. BAP1 mediates the transcriptional repression of SLC7A11, leading to cystine and GSH depletion. This DUB also promotes ACSL4 upregulation, which promotes the formation of lipid substrates whose peroxidation triggers ferroptosis. These two mechanisms might act synergistically to sensitize cells to ferroptosis by BAP1 (Fig. 1). However, it remains unclear how BAP1’s deubiquitinase activity can simultaneously repress *SLC7A11* and activate *ACSL4* expression. Zhang et al. proposed a model in which BAP1-mediated H2AK119ub deubiquitination at the SLC7A11 gene inhibits transcription initiation and elongation by reducing phosphorylation of serine 5 (S5-CTD) and serine 2 (S2-CTD) on the C-terminal domain of RNA Pol II [7]. Nevertheless, it is still unresolved how and in which context, BAP1 activity, generally associated with chromatin opening and gene activation, can result in transcriptional repression. It is plausible that cofactors such as ASXL1/2, which participate in both transcriptional activation and repression, play a key role in this dual functionality of BAP1. It is also possible that the global decrease in H2AK119ub following BAP1 re-expression in BAP1-deficient cells could lead to a dilution of PRC2 recruitment across the genome, thereby activating certain target genes [14]. Consequently, the repression of *SLC7A11* and the activation of *ACSL4* might result from a nonspecific redistribution of PRC2 on chromatin following BAP1 restoration. To further explore this possibility, it would have been informative to perform a ChIP-seq analysis for H3K27me3 and EZH2 (the catalytic subunit of PRC2) to compare their genomic distribution between BAP1-expressing and BAP1-deficient cells, as well as to assess changes at BAP1 target genes identified by the authors. Another limitation of this study is that the authors did not identify the transcription factors that might mediate the BAP1-dependent regulation of *ACSL4*. They used knockout cells for HIF1α, c-Myc, and STING/STAT1/IRF1 to assess their potential involvement and found that none of these factors affected *ACSL4* regulation by BAP1. However, it would have been interesting to examine other transcription factors such as SP1, which is known to directly regulate *ACSL4* expression and has been linked to BAP1 in some studies [15, 16].

Given that BAP1 also participates in apoptotic cell death [17], it would be of great interest to delineate the contexts in which BAP1 activates apoptosis versus ferroptosis. Under metabolic stress conditions, such as dysregulation of lipid or iron metabolism, BAP1 may preferentially trigger ferroptosis. Finally, since ferroptosis represents an emerging form of regulated cell death, identifying reliable in vivo setting where this form of cell death can be at play could have implications for treating neurodegenerative diseases and cancers. Indeed, Cañeque et al. recently demonstrated that lysosomal iron accumulation and activation initiate ferroptosis. They identified a small molecule, fentomycin-1, capable of specifically activating lysosomal iron and inducing ferroptosis. Furthermore, several CD44^high^ cancers, including sarcomas and pancreatic adenocarcinomas, exhibit high iron content, which confers strong metastatic potential and therapeutic resistance. Remarkably, fentomycin-1 selectively targeted these iron-rich cells, reducing tumor growth and extending survival in murine breast cancer models [18]. These findings suggest that ferroptosis could be therapeutically exploited in cancers with intrinsic ferroptotic vulnerabilities, particularly those characterized by lysosomal iron accumulation. In this context, BAP1-mutant tumors may represent especially suitable candidates for such approaches. A combined strategy involving SLC7A11 inhibition (to deplete cystine) and fentomycin-1-mediated ferroptosis activation could offer a promising treatment avenue for these cancers.
